# Investigating Molecular Interactions through Computational Modeling, Thermodynamic Analysis, and Acoustic Measurements of LiOTf in Aqueous TEGDME and DME Solutions at Different Temperatures

**DOI:** 10.1021/acsomega.4c07709

**Published:** 2024-12-24

**Authors:** Chitra Sharma, Harpreet Kaur, Abhinay Thakur, Akshay Sharma, Ramesh Chand Thakur, Harmanjit Singh Dosanjh, Vivek Pathania

**Affiliations:** † Department of Chemistry, School of Chemical Engineering and Physical Sciences, Lovely Professional University, Phagwara, Punjab 144411, India; ‡ Department of Chemistry, Himachal Pradesh University, Summer Hill, Shimla, Himachal Pradesh 171005, India; § Department of Chemistry, DAV College, Sector-10, Chandigarh 160011,India

## Abstract

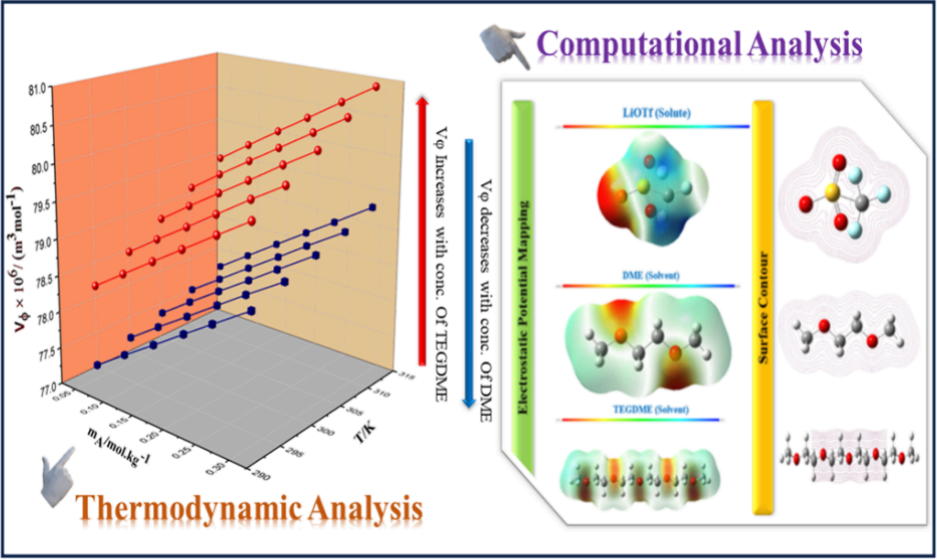

This study investigates solute–solvent interactions in ternary systems consisting of lithium trifluoromethanesulfonate (LiOTf) as the solute and tetraethylene glycol dimethyl ether (TEGDME) and 1,2-dimethoxyethane (DME) as solvents over a range of temperatures (293.15–313.15 K). A multidisciplinary approach involving computational modeling, thermodynamic analysis, and acoustic measurements was used to elucidate molecular-level dynamics. The positive *V*_ϕ_^0^ values in the thermodynamic analysis revealed the prevalence of solute–solvent interactions in the investigated ternary (LiOTf + H_2_O + DME/TEGDME) solutions. Hepler’s constant was determined to predict the structure maker/breaker behavior. Cyclic voltammetry analysis showed that TEGDME offers a higher electrochemical window (EW) of 1.36 V in 0.01 TEGDME and 1.40 V in 0.05 TEGDME compared with that of 1.25 V in 0.01 DME and 1.38 V in 0.05 DME, yielding favorable and comparable working EWs. DFT calculations using the B3LYP functional and 6-311++G(d,p) basis set provided insights into the electron-donating and -accepting properties of the molecules, showing higher reactivity for LiOTf. These findings present novel insights into ternary electrolyte systems, which hold the potential for applications in energy storage technologies.

## 1 Introduction

Energy storage devices play a pivotal role in modern energy systems, particularly in integrating renewable energy sources into the power grid.^1−3^ For example, in grid-scale applications, large battery storage systems such as lithium-ion batteries are utilized to store excess energy generated from renewable sources such as wind and solar during periods of low demand. This stored energy can then be discharged during peak demand periods or when renewable energy generation is low, helping to stabilize the grid and ensure a reliable power supply.^4−7^ Moreover, in transportation, electric vehicles (EVs) utilize energy storage in the form of lithium-ion batteries to store electrical energy for propulsion. The development of advanced battery technologies with higher energy density and faster charging capabilities is driving the adoption of EVs, reducing greenhouse gas emissions and dependence on fossil fuels in the transportation sector.^8−11^ Their ability to respond rapidly to fluctuations in supply and demand helps mitigate grid instability, ensuring reliable and resilient electricity delivery. Furthermore, energy storage devices facilitate load shifting, allowing electricity to be stored during off-peak hours and discharged during peak demand periods. This load management strategy optimizes grid operations, reduces the need for expensive peaker plants, and enhances overall efficiency.^12,13^ Additionally, these devices serve as crucial backup power sources during times of grid instability or outages, providing an uninterrupted electricity supply to essential services such as hospitals, emergency response systems, and telecommunications networks. Among the various energy storage options, electrochemical energy storage devices, such as batteries and supercapacitors, have garnered significant attention due to their high efficiency, scalability, and versatility. Electrolyte, a key component that facilitates ion transport between the electrodes, is majorly responsible for the performance and efficiency of electrochemical energy storage devices by enabling the storage and release of energy.^14,15^ Traditionally, electrolytes consist of lithium salts dissolved in organic solvent matrices. However, recent advancements in electrolyte design have led to the exploration of ternary electrolyte systems, wherein multiple solvents are combined with a lithium salt to enhance electrolyte properties such as conductivity, stability, and safety.^16,17^

This article adopts a multidisciplinary approach, integrating computational modeling, thermodynamic analysis, and acoustic measurements to delve into the complex behavior of electrolytes in energy storage devices. By combining these methodologies, the study aims to uncover the fundamental mechanisms governing electrolyte behavior and propose strategies for enhancing the performance and safety of such devices.^18,19^ The main focus of this investigation is lithium trifluoromethanesulfonate (LiOTf), which serves as the solute in ternary electrolyte systems. As a lithium salt, LiOTf undergoes dissociation in the solution, yielding lithium ions (Li^+^) and trifluoromethanesulfonate ions (OTf^–^). The Li^+^ ions play a pivotal role as primary charge carriers in electrochemical energy storage devices, traversing between the electrodes during charge and discharge cycles.^20,21^ LiOTf ensures a steady supply of Li^+^ ions within the electrolyte, facilitating the ion transport processes essential for energy storage. Additionally, tetraethylene glycol dimethyl ether (TEGDME) and 1,2-dimethoxyethane (DME) are commonly utilized as solvents in ternary electrolyte formulations. These solvents possess advantageous properties, including high dielectric constants, low viscosities, and wide electrochemical windows (EWs), rendering them conducive to efficient ion transport. TEGDME and DME not only aid in the dissolution of LiOTf but also serve as mediums for ion conduction within the energy storage device. Their presence ensures the smooth operation of charge and discharge processes, contributing to the overall efficiency and effectiveness of the energy storage system.^22,23^

Understanding molecular interactions is crucial for optimizing the design and performance of ternary electrolyte systems in energy storage devices.^24^ By elucidating the underlying mechanisms of molecular interactions, researchers can tailor electrolyte formulations to meet specific performance requirements, such as high conductivity, wide operating temperature range, and long cycle life.^25−28^ This study employs a multidisciplinary approach encompassing computational modeling, thermodynamic analysis, and acoustic measurements to characterize various interactions in ternary electrolyte systems,^29−31^ shedding light on previously unexplored aspects of ternary electrolyte systems.^32−34^ Furthermore, the focus on specific solute–solvent combinations, namely, LiOTf as the solute and TEGDME and DME as the solvents, adds novelty to the study. By emphasizing these particular components, the article aims to uncover unique insights into their interactions and their implications for energy storage device performance.^35−37^ Moreover, the investigation of solute–solvent interactions across a broad range of temperatures adds another dimension of novelty to the study. This temperature-dependent analysis may reveal temperature-sensitive phenomena that could impact the performance and stability of energy storage devices, thus offering valuable insights for future research and development efforts.^38,39^

## 2 Materials and Methods

### 2.1 Chemical Specifications

Table 1 provides a comprehensive overview of the chemicals utilized in the study, including their respective sources, CAS numbers, mass fraction purity, purification methods, and structures. Each of these specifications is crucial for ensuring the accuracy and reliability of the experimental results. The following chemicals sourced from Sigma-Aldrich with an average mass fraction purity of around 99% underwent purification through vacuum drying: LiOTf, DME, and TEGDME. The purification method specified for each chemical is particularly noteworthy as it ensures the removal of any impurities that could potentially interfere with the experimental outcomes by eliminating moisture and volatile contaminants.

**Table 1:** List of Chemicals Used, Their Source, CAS Number, Mass Fraction Purity, Purification Method, and Structure^a^

chemical	source	CAS number	mass fraction purity (%)	purification method
LiOTf	Sigma-Aldrich	33454-82-9	99.995	vacuum drying
DME	Sigma-Aldrich	110-71-4	>99.5	vacuum drying
TEGDME	Sigma-Aldrich	143-24-8	≥99	vacuum drying

a As specified by the supplier.

### 2.2 Density Analysis

The samples were weighed using a weighing balance (Sartorius CPA225D semimicro balance) with an accuracy of ±0.00001 g. The water for sample preparation had a precise conductance of <5 × 10^–6^ S·cm^–1^, obtained through a two-step filtration and ion exchange process using Millipore’s Milli-Q Academic water purification technology, followed by degassing. The density of the samples was evaluated using an Anton Paar DSA 5000 M, calibrated before each measurement set. Calibration involved measuring the density and speed of sound using dried air and deionized, triple-distilled water at 293.15 K with a precision of around ±1 × 10^–3^ kg m^–3^ for density. The device’s Peltier thermostat, with a temperature regulation accuracy of ±1 × 10^–3^ K, maintained stable conditions.

### 2.3 Speed of Sound Analysis

The Anton Paar DSA 5000 M was also utilized to measure the speed of sound in the samples. Ultrasonic velocity propagation was determined using a time approach, where a specimen placed between two piezoelectric ultrasonic transducers allowed for the transmission of 3 MHz sound waves. The instrument’s sensitivity enabled accurate measurements of acoustic values, with a precision of around ±1 × 10^–3^ kg m^–3^ for density and ±1 × 10^–2^ m s^–1^ for acoustic values.

### 2.4 Computational Analysis

In this study, we employed density functional theory (DFT) calculations to probe the intricate solute–solvent interactions within a ternary system composed of LiOTf as the solute and TEGDME and DME as the solvents. Herein, LiOTf was modeled as a molecule in its neutral state to elucidate its interaction with solvents TEGDME and DME. This modeling approach was chosen to capture the molecular-level dynamics and electronic properties of LiOTf in the ternary system. The computational framework utilized Gaussian software, a widely recognized tool in computational chemistry, and was performed using the B3LYP functional in conjunction with the 6-311G++(d,p) basis set. The inclusion of diffuse and polarization functions (denoted by “d” and “p”) allows for a more accurate representation of electron correlation effects and molecular interactions, essential for capturing the intricacies of solute–solvent interactions. This basis set is well-suited for accurately capturing the electronic structures and molecular properties of the involved species. To optimize the geometries of the solute and solvent molecules, we utilized the aforementioned basis set, ensuring convergence to stable and energetically favorable structures. Subsequently, various molecular descriptors were computed to comprehensively characterize the solute–solvent interactions. These descriptors include dipole moments, providing insights into the polarity of the molecules, and Mulliken charges, elucidating the distribution of electron density within the molecules. Additionally, the highest occupied molecular orbital (HOMO) and lowest unoccupied molecular orbital (LUMO) energies were determined to assess the electron-donating and -accepting capabilities of the species, respectively. Electrostatic potential maps and surface contours were also generated, offering a visual representation of the charge distribution and molecular surface topology. These calculations collectively enabled a detailed exploration of the molecular properties and solute–solvent interactions within the ternary system, facilitating a deeper understanding of its behavior and potential applications.

### 2.5 Cyclic Voltammetry (CV) Analysis

CV is a powerful and widely used electrochemical technique for studying the electrochemical properties of materials, particularly for determining the EW of solvents and electrolytes. In this study, CV analysis was conducted using a Metrohm Autolab PGSTAT204 multichannel potentiostat/galvanostat, which is a sophisticated and versatile electrochemical workstation.^19,40,41^ This equipment allows precise control and measurement of electrochemical processes, making it ideal for detailed studies of electrochemical behavior. In the CV analysis, a silver chloride (Ag/AgCl) reference electrode was used. The reference electrode consists of a silver wire coated with solid silver chloride immersed in a 3 M KCl solution. This standard configuration ensures stable and reproducible potential measurements during electrochemical testing.

In the analysis, solutions of DME and TEGDME were prepared along with these solvents containing various concentrations of LiOTf. The CV measurements were carried out at a scan rate of 100 mV/s, which is a typical rate that balances the sensitivity and resolution in detecting electrochemical processes. By cycling the potential between predetermined lower and upper limits and recording the corresponding current, CV plots (cyclic voltammograms) were obtained. These plots display the working electrode potential against the current, revealing the electrochemical activity within the tested potential range.^1,42^ The EW was identified by locating the potential values at the beginning and end of the flat current regions in the CV plots. These values represent the limits where only capacitive currents flow and no significant Faradaic reactions occur. The EW was calculated by subtracting the lower potential limit from the upper potential limit.

## 3 Results and Discussion

### 3.1 Density and Acoustic Measurements

The densities (ρ) of LiOTf in an aqueous solution of DME as well as TEGDME with concentrations of glyme *m* = (0.01, 0.03, and 0.05) mol·kg^–1^ and LiOTf concentrations ranging from (0.05 to 0.30) mol·kg^–1^ at *T* = (293.15, 298.15, 303.15, 308.15, and 313.15) K were ascertained in this study. Tables 2 and 6 show the measured density and acoustic values.

**Table 2:** Molality, Densities (ρ), and Apparent Molar Volumes (*V*_ϕ_) of LiOTf in Aqueous DME and Aqueous TEGDME Solutions at Various Temperatures, *P* = 0.1 MPa

*m*_A_^a^/(mol·kg^–^^1^)	ρ *×* 10^–3^/(kg·m^–3^)					*V*_ϕ_*×* 10^6^/(m^3^·mol^–1^)				
	*T* = 293.15 K	*T* = 298.15 K	*T* = 303.15 K	*T* = 308.15 K	*T* = 313.15 K	*T* = 293.15 K	*T* = 298.15 K	*T* = 303.15 K	*T* = 308.15 K	*T* = 313.15 K
LiOTf + 0.01 mol·kg^–1^DME										
0.00000	0.998273	0.996680	0.995411	0.993617	0.992051					
0.04999	1.002167	1.000548	0.999246	0.997421	0.995825	78.13	78.41	78.85	79.21	79.59
0.09983	1.006050	1.004406	1.003070	1.001214	0.999588	78.43	78.70	79.15	79.50	79.88
0.14999	1.009957	1.008288	1.006918	1.005031	1.003375	78.73	79.00	79.45	79.79	80.16
0.19951	1.013815	1.012120	1.010717	1.008800	1.007114	79.03	79.29	79.74	80.08	80.44
0.24983	1.017735	1.016015	1.014578	1.012629	1.010913	79.33	79.58	80.03	80.37	80.73
0.29986	1.021632	1.019886	1.018416	1.016436	1.014690	79.62	79.87	80.32	80.65	81.01
LiOTf + 0.03 mol·kg^–1^ DME										
0.00000	0.997518	0.996162	0.994835	0.992985	0.991648					
0.04993	1.001410	1.000032	0.998679	0.996799	0.995437	77.97	78.19	78.50	78.81	79.11
0.09855	1.005200	1.003801	1.002424	1.000515	0.999128	78.26	78.49	78.79	79.10	79.39
0.14993	1.009205	1.007783	1.006380	1.004440	1.003028	78.57	78.79	79.09	79.40	79.69
0.19831	1.012976	1.011533	1.010105	1.008136	1.006700	78.86	79.08	79.37	79.68	79.97
0.24855	1.016893	1.015427	1.013974	1.011974	1.010513	79.15	79.37	79.67	79.97	80.26
0.29962	1.020873	1.019386	1.017906	1.015876	1.014389	79.45	79.67	79.96	80.26	80.55
LiOTf + 0.05 mol·kg^–1^ DME										
0.00000	0.996974	0.995741	0.994188	0.992591	0.990711					
0.04979	1.000908	0.999650	0.998071	0.996450	0.994542	76.83	77.12	77.40	77.64	77.90
0.09983	1.004861	1.003578	1.001974	1.000328	0.998393	77.14	77.44	77.71	77.95	78.20
0.14981	1.008809	1.007501	1.005872	1.004201	1.002239	77.45	77.74	78.01	78.25	78.50
0.20215	1.012944	1.011610	1.009953	1.008257	1.006266	77.77	78.06	78.33	78.56	78.81
0.24599	1.016407	1.015051	1.013373	1.011655	1.009640	78.04	78.33	78.59	78.82	79.07
0.29951	1.020635	1.019253	1.017547	1.015803	1.013758	78.36	78.65	78.91	79.14	79.38
LiOTf + 0.01 mol·kg^–1^ TEGDME										
0.00000	0.998601	0.997162	0.995575	0.993951	0.992427					
0.04995	1.002542	1.001083	0.999476	0.997832	0.996287	77.19	77.36	77.51	77.66	77.83
0.09981	1.006476	1.004997	1.003370	1.001707	1.000142	77.50	77.67	77.82	77.96	78.13
0.14972	1.010414	1.008915	1.007268	1.005584	1.003999	77.80	77.97	78.12	78.26	78.43
0.19867	1.014276	1.012757	1.011091	1.009387	1.007782	78.10	78.27	78.41	78.55	78.72
0.24314	1.017784	1.016248	1.014564	1.012843	1.011219	78.37	78.54	78.68	78.82	78.98
0.29987	1.022261	1.020702	1.018995	1.017251	1.015604	78.71	78.87	79.02	79.15	79.32
LiOTf + 0.03 mol·kg^–1^ TEGDME										
0.00000	0.999412	0.997889	0.996373	0.994742	0.993179					
0.04992	1.003345	1.001797	1.000257	0.998598	0.997010	77.42	77.68	77.94	78.23	78.50
0.10109	1.007378	1.005805	1.004238	1.002551	1.000937	77.73	77.99	78.25	78.54	78.81
0.14978	1.011215	1.009617	1.008026	1.006313	1.004673	78.03	78.28	78.54	78.83	79.09
0.19995	1.015168	1.013545	1.011929	1.010188	1.008523	78.33	78.58	78.84	79.12	79.39
0.24328	1.018583	1.016938	1.015300	1.013536	1.011848	78.59	78.84	79.09	79.38	79.64
0.29950	1.023013	1.021340	1.019674	1.017878	1.016163	78.93	79.17	79.42	79.71	79.96
LiOTf + 0.05 mol·kg^–1^ TEGDME										
0.00000	1.000032	0.998702	0.997110	0.995511	0.993876					
0.04997	1.003930	1.002574	1.000955	0.999328	0.997666	78.31	78.60	78.90	79.20	79.50
0.09987	1.007822	1.006442	1.004795	1.003141	1.001451	78.61	78.90	79.20	79.50	79.79
0.14993	1.011727	1.010322	1.008647	1.006966	1.005248	78.91	79.19	79.49	79.79	80.08
0.19738	1.015428	1.013999	1.012299	1.010591	1.008848	79.19	79.47	79.76	80.06	80.35
0.24978	1.019515	1.018060	1.016331	1.014595	1.012822	79.50	79.78	80.07	80.36	80.65
0.29955	1.023397	1.021917	1.020160	1.018397	1.016597	79.79	80.06	80.35	80.64	80.93

a *m*_A_ denotes the molality of LiOTf in aqueous DME and aqueous TEGDME solutions. Standard uncertainty in the molality of LiOTf *u*_r_ (*m*_A_) is 1%. Standard uncertainty in the molality of DME and TEGDME *u*_r_ is 1.5%. Standard uncertainty in density, *u*(ρ) = ± 5 × 10^–3^ kg·m^–3^, temperature, *u*(*T*) = 0.001 K, and pressure, *u*(*p*) = 0.01 MPa.

#### 3.1.1 Thermophysical Parameters Derived from Density Measurements

Table 2 shows the increase of the density values of LiOTf as the concentration of Li salt in the aqueous solution of DME and aqueous TEGDME increases but drops when the temperature rises. Furthermore, with the increase in the concentration of DME, the density decreases, whereas in the case of LiOTf in TEGDME, the density values increase with the concentration of glyme.

##### 3.1.1.1 Apparent Molar Volume

**Figure 1:**
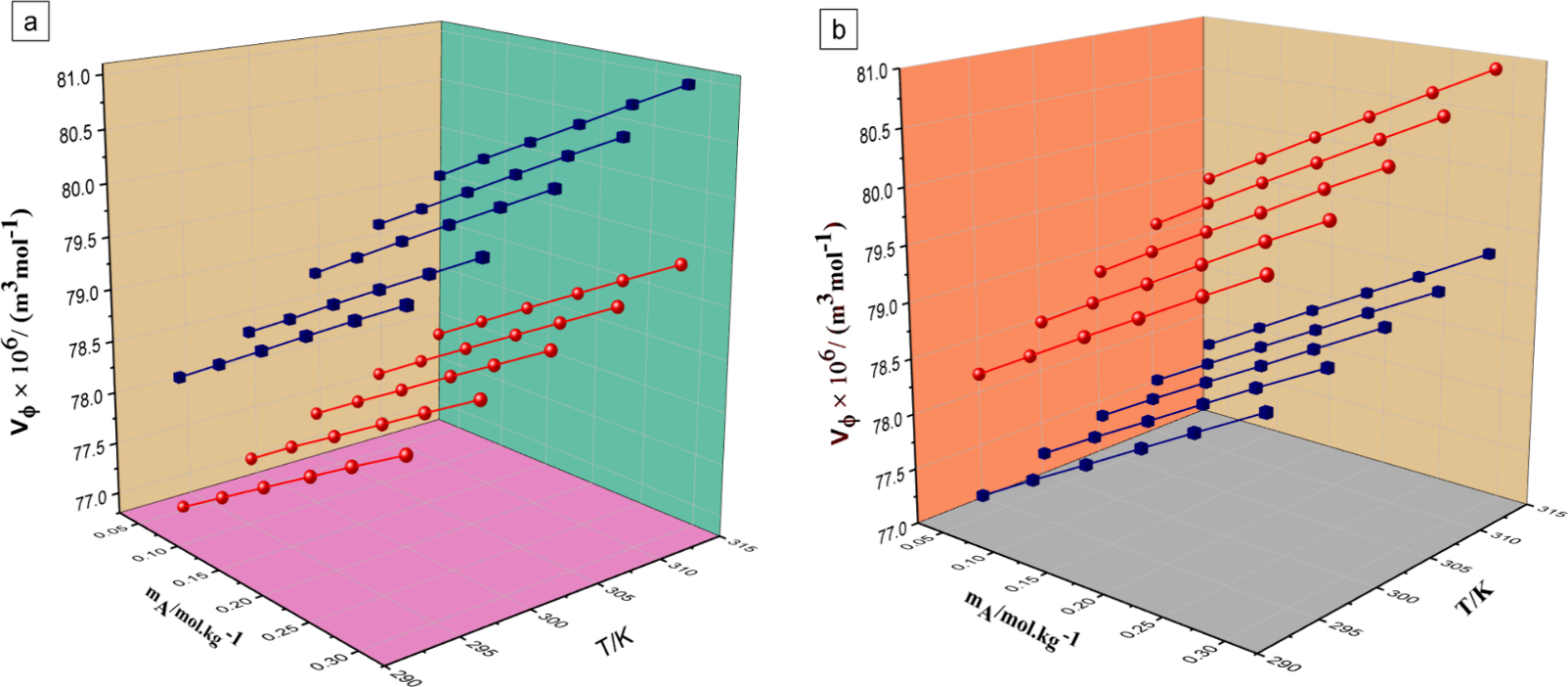
Plots of variation of apparent molar volume (*V*_ϕ_) of LiOTf in 0.01 mol·kg^–1^ (labeled as blue) and 0.05 mol·kg^–1^ (labeled as red) in (a) aqueous DME solution and (b) aqueous TEGDME solution at *T* = (293.15, 298.15, 303.15, 308.15, and 313.15 K).

The apparent molar volume (*V*_ϕ_) has been determined by utilizing the equation below:
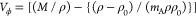
1Here, *M* and *m*_A_ denote the molar mass (kg·mol^–1^) and molality of LiOTf correspondingly, and ρ_0_ and ρ denote the densities (kg·m^–3^) of the solvent and solution. The resulting apparent molar volume values are listed in Table 2. In both cases, the *V*_ϕ_ values increase with rising temperature, which can be attributed primarily to thermal expansion effects. Analyzing the data, it is clear that *V*_ϕ_ values are greater at higher temperatures as the increase in temperature causes an increase in the thermal movement of molecules; hence, an increase in volume occurs. Moreover, the values of *V*_ϕ_ rise with the increase in the concentration of Li salt in the ternary solution (LiOTf + water + DME/TEGDME).^43^ Furthermore, it was observed that the values of LiOTf decrease with the increase in the concentration of DME, whereas they increase with the increase in the concentration of TEGDME. Figure 1 includes the diagrammatic representation of *V*_ϕ_ of LiOTf in 0.01 and 0.05 mol·kg^–1^ in (a) an aqueous DME solution and (b) an aqueous TEGDME solution at *T* = (293.15, 298.15, 303.15, 308.15, and 313.15) K.

##### 3.1.1.2 Partial Molar Volume

The partial molar volume at infinite dilution (*V*_ϕ_^0^) was calculated utilizing Masson’s least-squares fitting approach in eq 2:
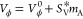
2Here, *V*_ϕ_^0^ denotes the partial molar volume.

*V*_ϕ_^0^ depicts the solute–solvent interactions, assuming that the solute molecules are apart at infinite dilution, and hence, no solute–solute interactions exist. The positive *V*_ϕ_^0^ value (Table 3) proves the occurrence of solute–solvent interactions in ternary systems. The electrostriction, which decreases as the temperature increases, could be responsible for the increase of *V*_ϕ_^0^ values with temperature.^44^ Some solvation molecules emerge at the solute’s loose solvation layers and penetrate the bulk solution sequentially. The water molecules that enclose LiOTf are compressed by the electric field induced by their head groups. As a result, increased apparent molar volume values in the ternary (LiOTf + H_2_O + DME/TEGDME) solutions indicate the prevalence of solute–solvent interactions.^4^ Furthermore, in the case of Li salt in DME, the *V*_ϕ_^0^ values decrease with an increase in the concentration of the aqueous solvent. In contrast, the *V*_ϕ_^0^ values of LiOTf in TEGDME increase with the concentration of TEGDME.

**Table 3:** Partial Molar Volumes (*V*_ϕ_^0^) of LiOTf in Aqueous DME and Aqueous TEGDME Solutions at Various Temperatures

*m*_B_ (mol·kg^–1^)^a^	*V*_ϕ_^0^ × 10^6^/(m^3^·mol^–1^)				
	*T* = 293.15 K	*T* = 298.15 K	*T* = 303.15 K	*T* = 308.15 K	*T* = 313.15 K
LiOTf in aqueous DME					
0.00	80.44 (±0.003)	79.85 (±0.006)	79.34 (±0.003)	78.59 (±0.003)	77.89 (±0.013)
0.01	77.84 (±0.003)	78.12 (±0.003)	78.56 (±0.006)	78.93 (±0.003)	79.31 (±0.003)
0.03	77.67 (±0.003)	77.90 (±0.003)	78.21 (±0.003)	78.53 (±0.003)	78.83 (±0.003)
0.05	76.53 (±0.003)	76.82 (±0.006)	77.11 (±0.003)	77.35 (±0.003)	77.61 (±0.003)
LiOTf in aqueous TEGDME					
0.01	76.89 (±0.003)	77.06 (±0.003)	77.22 (±0.003)	77.36 (±0.003)	77.54 (±0.003)
0.03	77.12 (±0.003)	77.38 (±0.003)	77.64 (±0.003)	77.94 (±0.003)	78.21 (±0.003)
0.05	78.01 (±0.003)	78.31 (±0.003)	78.61 (±0.003)	78.92 (±0.003)	79.22 (±0.003)

a *m*_B_ is the molality of aqueous DME and aqueous TEGDME solutions.

The partial molar volume of transfer, Δ*V*_ϕ_^0^, of LiOTf from water to the aqueous solvent (DME/TEGDME), at infinite dilution, has been evaluated by employing the following relation:
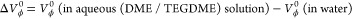
3

Δ*V*_ϕ_^0^ values given in Table 4 signify solute–solvent interactions particularly; thus, the contribution from solute–solute interactions is considered to be negligible. The co-sphere overlap model is useful in predicting the possibility of shrinkage or expansion in the volume as a consequence of solute–solvent interactions.^45^ The negative values of the transfer volumes at lower temperatures suggest that hydrophobic–hydrophobic interactions are predominant and can be attributed to the increase in electrostriction. However, at elevated temperatures, ion–ion interactions are significant as here higher Δ*V*_ϕ_^0^ values are observed because the H_2_O molecules earlier existing near hydrophilic centers are now discharged to the bulk that is highly compressible in comparison to the electrostricted region.

**Table 4:** Partial Molar Volume of Transfer, Δ*V*_ϕ_^0^× 10^6^/(m^3^ mol^–1^), of LiOTf in Aqueous DME and Aqueous TEGDME Solutions at Various Temperatures

*m*_B_^a^(mol·kg^–1^)	Δ*V*_ϕ_^0^				
	*T* = 293.15 K	*T* = 298.15 K	*T* = 303.15 K	*T* = 308.15 K	*T* = 313.15 K
LiOTf in aqueous DME					
0.01	–2.60	–1.73	–0.78	0.34	1.42
0.03	–2.77	–1.95	–1.14	–0.06	0.93
0.05	–3.91	–3.04	–2.24	–1.24	–0.28
LiOTf in aqueous TEGDME					
0.01	–3.55	–2.79	–2.13	–1.23	–0.35
0.03	–3.32	–2.48	–1.70	–0.65	0.32
0.05	–2.43	–1.55	–0.73	0.33	1.33

a *m*_B_ is the molality of aqueous DME and aqueous TEGDME solutions.

##### 3.1.1.3 Temperature Dependence of Partial Molar Volume

Temperature has a significant impact on the interactions in a system. The influence of temperature on apparent molar volume (*V*_ϕ_^0^) (at infinite dilution) has been evaluated utilizing the polynomial equation below:
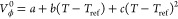
4Here, *T*_ref_ is the reference temperature (298.15 K), whereas the empirical constants are *a*, *b*, and *c*. Table 5 enlists the values of these empirical constants. Apart from *c* at *m*_DME_= 0.05 mol·kg^–1^ and *m*_TEGDME_= 0.01 mol·kg^–1^ for LiOTf, wherein empirical constant *c* has minor negative values, all empirical constants are positive.^46^

**Table 5:** Values of Empirical Parameters Determined from Eq 3 for LiOTf in Aqueous DME and Aqueous TEGDME Solutions

*m*_B_^a^/(mol·kg^–1^)	*a* × 10^6^/(mol·kg^–1^)	*b* × 10^6^/(m^3^·mol^–1^·K)	*c* × 10^6^/(m^3^·mol^–1^·K^–2^)	*R*^2^	ARD (σ)
LiOTf in aqueous DME					
0.01	78.17	0.071	0.0003	0.9999	0.0013
0.03	77.92	0.054	0.0004	0.9999	0.0007
0.05	76.82	0.056	–0.0003	0.9999	0.0003
LiOTf in aqueous TEGDME					
0.01	77.05	0.032	–0.00001	0.9999	0.0003
0.03	77.38	0.053	0.0002	0.9999	0.0003
0.05	78.31	0.06	0.0001	0.9999	0.0001

a *m*_B_ is the molality of aqueous DME and aqueous TEGDME solutions.

The experimental values of *V*_ϕ_^0^ deviate from the theoretical values. The ARD (σ) deviations could be determined by employing the equation below:
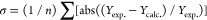
5where *Y* = *V*_ϕ_^0^. Table 5 summarizes the ARD data, demonstrating a strong fit to the experimental data for the ternary compositions studied.

Furthermore, partial molar expansibilities, ϕ_E_^0^, were used to confirm the presence of solute–solvent interactions in the mixtures under study by utilizing the equation given below:
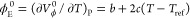
6

For all quantities of DME and experimental temperatures, the positive partial molar expansibilities (given in Table 6) are consistent with the expected thermal expansion behavior of the solution as the temperature increases and should not be directly interpreted as evidence of unusual solute–solvent interactions. Additionally, the packing effect verifies the occurrence of substantial interactions between LiOTf and DME as well as LiOTf in TEGDME with the reported positive values of ϕ_E_^0^.

**Table 6:** Limiting Apparent Molar Expansibilities (ϕ_E_^0^) of LiOTf in Aqueous Solutions of DME and TEGDME at Various Temperatures

*m*_B_^a^/(mol·kg^–1^)	ϕ_*E*_^0^ × 10^6^/(m^3^·mol^–1^·mol^–1^·K^–1^)					(∂ϕ_E_^0^/∂*T*)
	*T* = 293.15 K	*T* = 298.15 K	*T* = 303.15 K	*T* = 308.15 K	*T* = 313.15 K	
LiOTf in aqueous DME						
0.01	0.0680	0.0714	0.0748	0.0782	0.0816	0.00068
0.03	0.0501	0.0544	0.0587	0.0629	0.0672	0.00086
0.05	0.0594	0.0566	0.0538	0.0510	0.0482	–0.00056
LiOTf in aqueous TEGDME						
0.01	0.0320	0.0319	0.0318	0.0317	0.0316	–0.00002
0.03	0.0519	0.0535	0.0550	0.0566	0.0581	0.00031
0.05	0.0596	0.0601	0.0606	0.0610	0.0615	0.00010

a *m*_B_ is the molality of aqueous DME and aqueous TEGDME solutions.

In a mixed solvent solution, the Hepler constant contributes to evaluating the solute’s potential to function as a structure breaker/maker. Hepler’s thermodynamic equation is written as follows:^47^
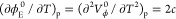
7

Positive and negative values of (∂ϕ_E_^0^/∂*T*)_p_ have been reported for the ternary systems investigated (LiOTf + H_2_O + DME; given in Table 6). The sign of (∂ϕ_E_^0^/∂*T*) could be used to evaluate the function of a solute as a structure maker/breaker.^48,49^ Structure makers have positive values of (∂ϕ_E_^0^/∂*T*) and minor negative values of (∂ϕ_E_^0^/∂*T*), while structure breaker solutes have negative values. In aqueous solutions, *m*_DME/TEGDME_ = (0.01, 0.03, and 0.05) mol·kg^–1^, and LiOTf behaves as structure makers.

### 3.2 Acoustic Parameters Derived from Speed of Sound (Acoustic) Measurements

The acoustic measurements provide valuable insights into the physical properties and interactions within the solution. Table 7 shows the sound speed of LiOTf in *m*_DME/TEGDME_ = (0.01, 0.03, and 0.05) mol·kg^–1^ of aqueous solutions at the above-specified temperatures.

**Table 7:** Values of Ultrasonic Speed (*u*) and Apparent Molar Isentropic Compressibility (*K*_ϕ,s_) of LiOTf in Aqueous DME and Aqueous TEGDME Solutions at Various Temperatures, *P* = 0.1 MPa

*m*_A_^a^/(mol·kg-^1^)	*u*/(m·s^–1^)					*K*_ϕ_,_s_× 10^6^(m^3^·mol^–1^·GPa^–1^)				
	*T* = 293.15 K	*T* = 298.15 K	*T* = 303.15 K	*T* = 308.15 K	*T* = 313.15 K	*T* = 293.15 K	*T* = 298.15 K	*T* = 303.15 K	*T* = 308.15 K	*T* = 313.15 K
LiOTf + 0.01 mol·kg^–1^ DME										
0.00000	1483.54	1497.11	1509.66	1520.66	1529.20					
0.04999	1484.81	1498.23	1510.64	1521.49	1529.90	–15.87	–13.26	–10.81	–8.52	–6.43
0.09983	1486.09	1499.34	1511.61	1522.32	1530.59	–15.96	–13.37	–10.91	–8.62	–6.55
0.14999	1487.37	1500.47	1512.59	1523.15	1531.29	–16.07	–13.48	–11.03	–8.74	–6.68
0.19951	1488.63	1501.57	1513.56	1523.98	1531.99	–16.17	–13.59	–11.14	–8.86	–6.81
0.24983	1489.91	1502.70	1514.54	1524.82	1532.69	–16.28	–13.70	–11.26	–8.98	–6.93
0.29986	1491.19	1503.82	1515.52	1525.65	1533.39	–16.38	–13.81	–11.37	–9.10	–7.05
LiOTf + 0.03 mol·kg^–1^ DME										
0.00000	1484.68	1498.35	1510.19	1520.69	1529.69					
0.04993	1485.43	1499.02	1510.78	1521.20	1530.12	–9.38	–8.03	–6.47	–5.11	–3.73
0.09855	1486.15	1499.68	1511.35	1521.70	1530.53	–9.53	–8.16	–6.60	–5.24	–3.86
0.14993	1486.92	1500.37	1511.95	1522.22	1530.97	–9.66	–8.28	–6.73	–5.37	–3.99
0.19831	1487.64	1501.02	1512.51	1522.72	1531.39	–9.79	–8.40	–6.85	–5.49	–4.11
0.24855	1488.39	1501.70	1513.10	1523.23	1531.82	–9.91	–8.53	–6.98	–5.62	–4.24
0.29962	1489.15	1502.39	1513.70	1523.76	1532.25	–10.04	–8.65	–7.10	–5.74	–4.37
LiOTf + 0.05 mol·kg^–1^ DME										
0.00000	1485.96	1499.43	1512.31	1521.74	1530.81					
0.04979	1486.51	1499.92	1512.74	1522.09	1531.09	–7.98	–6.80	–5.57	–4.25	–3.00
0.09983	1487.06	1500.42	1513.17	1522.44	1531.37	–8.11	–6.92	–5.69	–4.37	–3.14
0.14981	1487.60	1500.91	1513.60	1522.79	1531.64	–8.23	–7.05	–5.81	–4.50	–3.26
0.20215	1488.18	1501.43	1514.05	1523.16	1531.94	–8.36	–7.18	–5.94	–4.63	–3.40
0.24599	1488.66	1501.86	1514.43	1523.47	1532.18	–8.47	–7.28	–6.05	–4.73	–3.50
0.29951	1489.25	1502.39	1514.89	1523.84	1532.48	–8.59	–7.41	–6.17	–4.86	–3.63
LiOTf + 0.01 mol·kg^–1^ TEGDME										
0.00000	1484.54	1497.38	1509.6	1521.1	1530.70					
0.04995	1485.76	1498.42	1510.37	1521.60	1531.10	–16.13	–13.26	–9.84	–6.61	–3.36
0.09981	1486.98	1499.46	1511.15	1522.10	1531.50	–16.19	–13.38	–9.97	–6.73	–3.51
0.14972	1488.20	1500.51	1511.92	1522.60	1531.89	–16.29	–13.50	–10.09	–6.85	–3.64
0.19867	1489.40	1501.53	1512.68	1523.09	1532.28	–16.38	–13.61	–10.20	–6.97	–3.77
0.24314	1490.49	1502.46	1513.37	1523.53	1532.64	–16.47	–13.71	–10.30	–7.08	–3.88
0.29987	1491.88	1503.64	1514.25	1524.10	1533.09	–16.58	–13.83	–10.43	–7.21	–4.02
LiOTf + 0.03 mol·kg^–1^ TEGDME										
0.00000	1487.46	1499.90	1511.73	1522.89	1532.40					
0.04992	1488.63	1501.20	1512.54	1523.46	1532.72	–16.06	–12.93	–9.78	–6.32	–2.98
0.10109	1489.84	1502.53	1513.38	1524.05	1533.04	–16.12	–13.08	–9.87	–6.49	–3.15
0.14978	1490.98	1503.80	1514.18	1524.61	1533.35	–16.22	–13.20	–9.98	–6.62	–3.29
0.19995	1492.16	1505.10	1514.99	1525.18	1533.66	–16.32	–13.31	–10.09	–6.75	–3.42
0.24328	1493.18	1506.23	1515.70	1525.68	1533.94	–16.40	–13.40	–10.19	–6.86	–3.53
0.29950	1494.50	1507.69	1516.62	1526.32	1534.29	–16.51	–13.52	–10.32	–6.99	–3.67
LiOTf + 0.05 mol·kg^–1^ TEGDME										
0.00000	1490.09	1502.85	1513.73	1524.89	1534.71					
0.04997	1491.31	1503.87	1514.56	1525.51	1534.99	–14.86	–11.87	–8.94	–6.05	–2.91
0.09987	1492.53	1504.88	1515.38	1526.13	1535.28	–14.99	–11.95	–9.10	–6.21	–3.08
0.14993	1493.75	1505.90	1516.21	1526.75	1535.56	–15.11	–12.05	–9.24	–6.35	–3.23
0.19738	1494.91	1506.86	1517.00	1527.34	1535.83	–15.21	–12.15	–9.36	–6.47	–3.36
0.24978	1496.19	1507.93	1517.87	1527.99	1536.13	–15.33	–12.27	–9.49	–6.61	–3.49
0.29955	1497.41	1508.94	1518.69	1528.61	1536.41	–15.43	–12.38	–9.61	–6.73	–3.62

a *m*_A_ denotes the molality of LiOTf in an aqueous DME medium and an aqueous TEGDME solution. Standard uncertainty in the molality of LiOTf *u*_r_(*m*_A_) is 1%. Standard uncertainty in the molality of DME and TEGDME *u*_r_ is 1.5%. Standard uncertainty in acoustic, *u*(*u*)= 0.5 m·s^–1^, *u*(*T*) = 0.001 K, and pressure, *u*(*p*) = 0.01 MPa.

#### 3.2.1 Apparent Molar Isentropic Compressibility

The apparent molar isentropic compressibility (*K*_ϕ,s_) can be determined by utilizing the equation below:
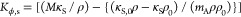
8where κ_S_ denotes solution isentropic compressibility and κ_S,0_ denotes pure solvent isentropic compressibility. The molality of the solute is *m*_A_, the molar mass of LiOTf and (solute) is *M*, and the density values of the solute and solvent are ρ and ρ_0_ correspondingly. κ_S_ can be calculated using the Laplace–Newton equation (eq 9)
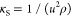
9where *u* and ρ refer to the density and acoustic values of the given solution.

**Figure 2:**
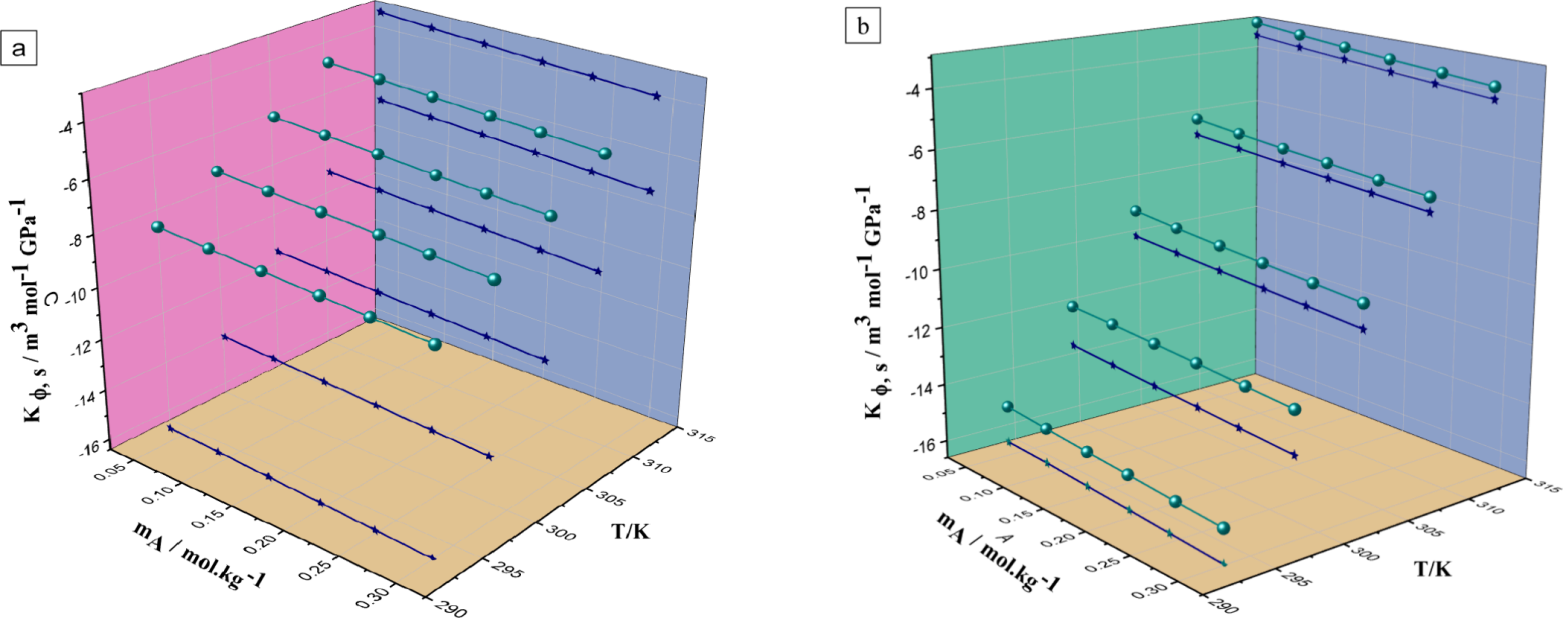
Plots of variation of apparent molar isentropic compressibility (*K*_ϕ,s_) of LiOTf in 0.01 mol·kg^–1^ (labeled as blue) and 0.05 mol·kg^–1^ (labeled as red) in (a) aqueous DME solution and (b) aqueous TEGDME solution at *T* = (293.15, 298.15, 303.15, 308.15, and 313.15 K).

The values of *K*_ϕ,s_ determined employing this method (eq 8) have been indexed in Table 7 and represented graphically in Figure 2.

*K*_ϕ,s_ is negative at all of the aqueous DME and aqueous TEGDME concentrations and temperatures. As the molality of LiOTf as well as DME and TEGDME increases and the temperature increases, the *K*_ϕ,s_ values of LiOTf ascend. Furthermore, as the concentration of the solvent increases, *K*_ϕ,s_ decreases. The occurrence of solute–solvent interactions is confirmed by the negative *K*_ϕ,s_ values. Negative values of *K*_ϕ,s_ indicate that H_2_O molecules in the bulk solution are much more deformable than those near the solute. Because of the charge on the ions, the electrostricted H_2_O molecules are mainly compacted. The electrostriction reduces as the temperature rises, and some H_2_O molecules are discharged from the hydration sphere into the bulk solution, resulting in less conformational distortion of water and showing a reduced regulating influence by the solute on the solvent. As *V*_ϕ_^0^ is defined at infinite dilution, it reflects solute–solvent interactions in the absence of significant solute–solute interactions. Therefore, the observed *V*_ϕ_^0^ values confirm the typical behavior expected in such dilute systems.^50^

#### 3.2.2 Partial Molar Isentropic Compressibility

Partial molar isentropic compressibility (*K*_ϕ,s_^0^) indicates the existence of (solute–solvent) interactions. The following equation calculates the variation of *K*_ϕ,s_^0^ with the molality of the solute:
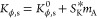
10

The molality of LiOTf utilized in an aqueous DME solution is denoted by *m*_A_. Table 8 shows the calculated values of *K*_ϕ,s_^0^ of the ternary systems. While raising the temperature, *K*_ϕ,s_^0^ decreases because water molecules are firmly bound to solute molecules at low temperatures, but as the temperature rises, the electrostriction between them reduces, and some H_2_O molecules are discharged into the bulk solution. As a result, partial molar isentropic compressibility inference suggests that in this investigation, solute–solvent interactions are predominant.^51^*K*_ϕ,s_^0^ deduces that the solvent molecules are bound to the solute molecules, which enhances the interactions between the ions.

**Table 8:** Partial Molar Isentropic Compressibility (*K*_ϕ,s_^0^) of LiOTf in an Aqueous Solution of DME and Aqueous TEGDME at Various Temperatures

*m*_B_^a^/(mol·kg^–1^)	*K*_ϕ,s_^0^*×*10^6^/(m^3^·mol^–1^·GPa^–1^)				
	*T* = 293.15 K	*T* = 298.15 K	*T* = 303.15 K	*T* = 308.15 K	*T* = 313.15 K
LiOTf in aqueous DME					
0.01	–15.77 (±0.005)	–13.15 (±0.003)	–10.69 (±0.005)	–8.39 (±0.004)	–6.3 (±0.002)
0.03	–9.26 (±0.01)	–7.91 (±0.003)	–6.35 (±0.004)	–4.99 (±0.002)	–3.61 (±0.002)
0.05	–7.86 (±0.004)	–6.68 (±0.002)	–5.45 (±0.002)	–4.13 (±0.002)	–2.88 (±0.002)
LiOTf in aqueous TEGDME					
0.01	–16.02 (±0.01)	–13.16 (±0.004)	–9.73 (±0.003)	–6.49 (±0.003)	–3.24 (±0.01)
0.03	–15.95 (±0.01)	–12.83 (±0.01)	–9.66 (±0.007)	–6.21 (±0.01)	–2.87 (±0.01)
0.05	–14.76 (±0.007)	–11.75 (±0.01)	–8.83 (±0.01)	–5.93 (±0.01)	–2.79 (±0.01)

a *m*_B_ is the molality of aqueous DME and aqueous TEGDME solutions.

#### 3.2.3 Pair and Triplet Interaction Coefficients

Pair interaction coefficients (*V*_AB_, *K*_AB_) and triplet interaction coefficients (*V*_ABB_, *K*_ABB_) assist in the interpretation of the interactions existing in the solvation sphere. These parameters represent divergence in the properties because of the interactions between two or more solute molecules. In order to determine the interaction coefficients, McMillan and Mayer put forward a theory that was revised further by Friedman and Krishanan.^52^ The interaction coefficients are calculated using the succeeding equations:
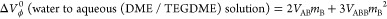
11
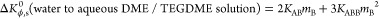
12Here, A stands for LiOTf and B represents DME/TEGDME, *m*_B_ is the concentration of the aqueous solvent. *V*_AB_ and *V*_ABB_ are volumes, whereas *K*_AB_ and *K*_ABB_ are isentropic compressions signifying pair and triplet interaction coefficients. The values of the interaction coefficients are listed in Table 9. From the results obtained, it is clear that at low temperatures, *V*_ABB_ > *V*_AB_ and *K*_ABB_ > *K*_AB_, whereas at high temperatures, *V*_AB_ > *V*_ABB_ and *K*_AB_ > *K*_ABB._. The higher values of *V*_AB_ and *K*_AB_ show that the interactions between LiOTf and DME/TEGDME are mainly pairwise.

**Table 9:** Pair Interaction Coefficients (*V*_AB,_*K*_AB_) and Triplet Interaction Coefficients (*V*_ABB,_*K*_ABB_) of LiOTf in an Aqueous Solution of DME and Aqueous TEGDME at Various Temperatures

*T*/(K)	*V*_AB_ × 10^6^/(m^3^ mol^–2^ kg)	*V*_ABB_ × 10^6^/(m^3^ mol^–3^ kg^2^)	*K*_AB_ × 10^6^/(m^3^ mol^–2^ kg GPa^–1^)	*K*_ABB_ × 10^6^/(m^3^ mol^–3^ kg^2^ GPa^–1^)
LiOTf in aqueous DME				
293.15	–88.54	686.86	–54.60	1339.28
298.15	–57.34	378.41	8.98	563.70
303.15	–23.53	23.87	73.06	–211.86
308.15	18.78	–418.07	129.73	–868.63
313.15	58.43	–829.71	178.66	–1444.61
LiOTf in aqueous TEGDME				
293.15	–139.67	1571.06	–158.01	1753.57
298.15	–110.08	1287.03	–73.17	942.69
303.15	–83.57	1037.52	–4.37	914.87
308.15	–47.18	686.23	199.97	–1869.02
313.15	8.88	131.67	323.47	–3192.49

### 3.3 DFT Analysis

To unveil critical insights into the behavior and performance of molecules within the ternary system, featuring LiOTf as the solute and TEGDME alongside DME as the solvents, the researchers employed advanced computational techniques. Specifically, a series of equations grounded in Koopmans’ theorem were utilized to calculate essential constraints such as the ionization potential (IP), electron affinity (EA), electronegativity (χ), hardness (η), and softness (σ).^53−55^ These calculations served as a pivotal means to discern the intrinsic properties and characteristics of the molecular constituents, shedding profound light on their interlinkages and interactions within the ternary environment. By leveraging these computational methodologies, the study aimed to unravel the intricate dynamics governing solute–solvent interactions, thus providing invaluable insights into the overall behavior and efficacy of the ternary system for its intended applications.
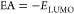
13
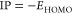
14
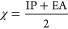
15
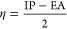
16
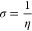
17

**Figure 3:**
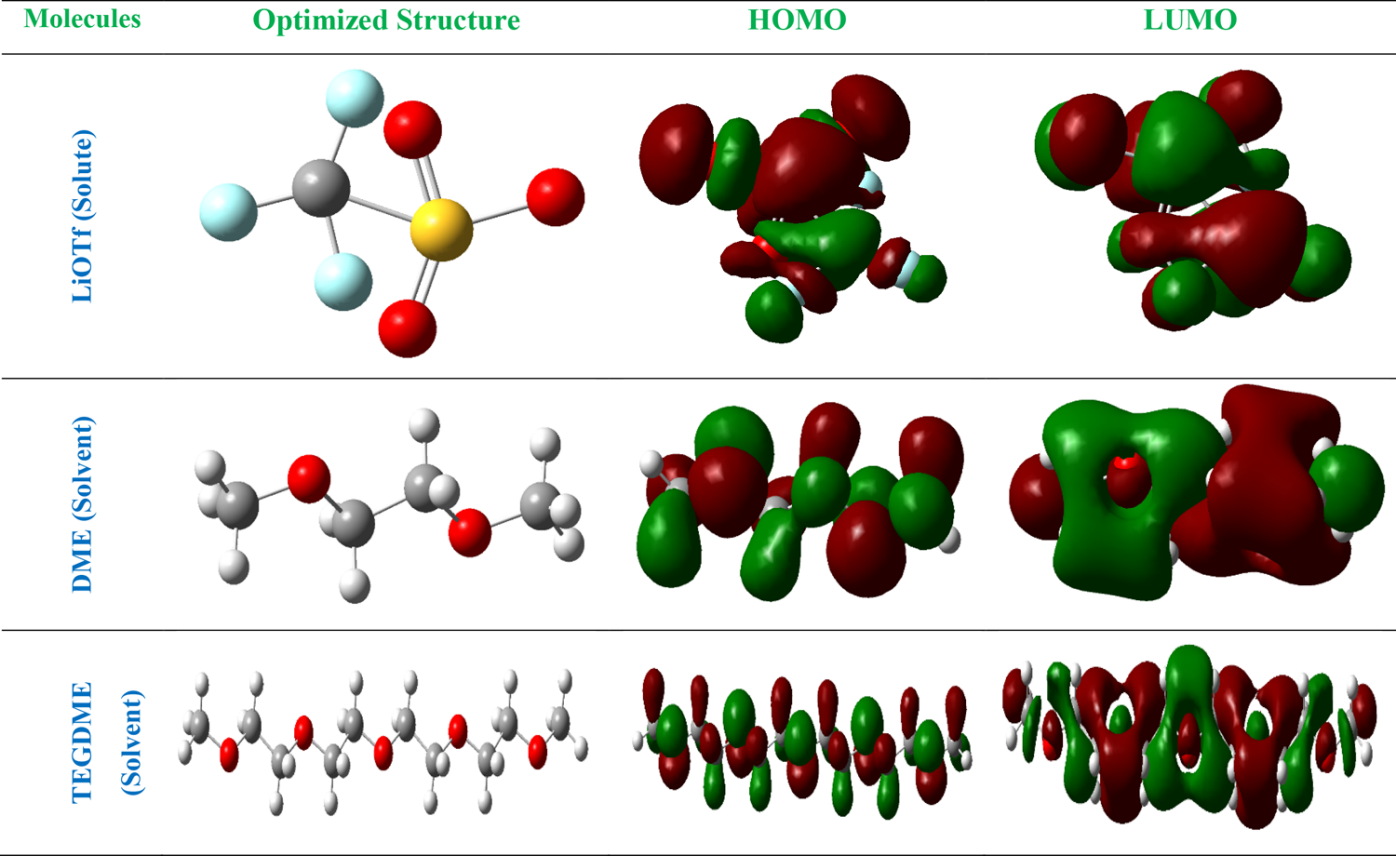
Optimized, HOMO, and LUMO structures of the molecules present in the ternary system.

Figure 3 visually represents these structures, providing insight into the spatial arrangement and electronic properties of the molecules. For LiOTf, the optimized structure reveals its molecular conformation, while the HOMO and LUMO energies indicate its electron-donating and -accepting capabilities, respectively. Similarly, the optimized structures of DME and TEGDME offer insights into their molecular conformations and spatial arrangements within the ternary system. The HOMO and LUMO energies provide information about their reactivity and potential for electron transfer processes, which are crucial for understanding their interactions with LiOTf and each other. This information is instrumental in elucidating the behavior and performance of the ternary system for various applications, such as electrolytes in batteries or solvents in chemical processes.

The quantum chemical descriptors presented in Table 10 offer valuable insights into the electronic structure and reactivity of the solute (LiOTf) and solvents (DME and TEGDME) within the ternary system. The *E*_HOMO_ and *E*_LUMO_ energies indicate the electron-donating and -accepting capabilities of the molecules, respectively. The *E*_HOMO_ and *E*_LUMO_ energies for LiOTf (−0.32536 and −0.02856 eV, respectively) suggest that it has a higher tendency to donate and accept electrons compared to the solvent molecules. DME and TEGDME have higher *E*_HOMO_ and *E*_LUMO_ energies, indicating that they are more likely to act as electron donors in interactions with LiOTf. This implies a strong potential for interaction with both solvents. The energy gap between *E*_HOMO_ and *E*_LUMO_ (Δ*E*) reflects the stability and reactivity of the molecules. LiOTf has a greater Δ*E* compared to the solvent molecules, indicating that it is less reactive than the solvents and has a lower potential for electron transfer processes.

**Table 10:** Quantum Chemical Descriptors of the Molecules Present in the Ternary System

molecules	*E*_HOMO_ (eV)	*E*_LUMO_ (eV)	Δ*E* (eV)	IP (eV)	EA (eV)	μ (D)	χ (eV)	η (eV)	σ (eV^–1^)	electronic energy (Hartree)
LiOTf (solute)	–0.32536	–0.02856	0.2968	0.32536	0.02856	3.622472	0.1484	0.17696	5.650994575	–950.804169
DME (solvent)	–0.24765	–0.07233	0.17532	0.24765	0.07233	0	0.08766	0.15999	6.250390649	–308.759959
TEGDME (solvent)	–0.24906	–0.05367	0.19539	0.24906	0.05367	1.678106	0.097695	0.151365	6.606547088	–770.108742

The IP and EA values for LiOTf (IP = 0.32536 eV and EA = 0.02856 eV) indicate a lower tendency to lose electrons, respectively. Ionization potential (IP) is the energy required to remove an electron from a molecule, and a higher IP value reflects greater difficulty in losing an electron, meaning that LiOTf is less likely to donate electrons. Similarly, a lower electron affinity (EA) implies that LiOTf has a reduced tendency to gain electrons. In comparison, the solvent molecules have even lower IP and EA values, suggesting that they are less resistant to electron transfer processes. Hence, while LiOTf may act as both an electron donor and an electron acceptor, its capacity for electron donation is relatively lower, with electron acceptance also being limited. The dipole moment quantifies the polarity of the molecules. LiOTf exhibits a relatively high dipole moment (μ = 3.622472 D) compared to the solvent molecules (μ ≈ 0–1.678106 D), indicating stronger polarity. This suggests that LiOTf may form stronger dipole–dipole interactions with the solvent molecules, influencing their solvation behavior and overall interaction. In the case of electronegativity, electronegativity reflects the ability of a molecule to attract electrons. LiOTf has a higher electronegativity (χ = 0.1484 eV) compared to the solvent molecules (χ ≈ 0.08766 eV), indicating stronger electron-attracting capabilities. This suggests that LiOTf may induce polarization in the solvent molecules, leading to enhanced solute–solvent interactions.

Furthermore, global hardness represents the resistance of a molecule to electron flow, while chemical softness reflects the ease of electron transfer. LiOTf exhibits relatively lower global hardness and higher chemical softness compared with the solvent molecules, indicating higher reactivity and potential for electron transfer processes. LiOTf exhibits lower global hardness (η ≈ 0.17696 eV) and higher chemical softness (σ ≈ 5.650994575–6.606547088 eV^–1^) compared to the solvent molecules. This indicates a higher reactivity and potential for electron transfer processes in LiOTf–solvent interactions. This implies that LiOTf may readily undergo interactions with the solvent molecules, leading to solvation and the formation of stable complexes.

Based on the quantum chemical descriptors provided in Table 10, it is inferred that TEGDME likely exhibits stronger interactions with LiOTf compared to DME within the ternary system. This conclusion is supported by several factors. First, TEGDME demonstrates a higher energy gap (Δ*E* = 0.19539 eV) compared to DME, suggesting lower reactivity and potentially stronger stability. Additionally, TEGDME boasts a dipole moment (μ = 1.678106 D) higher than that of DME, indicating stronger polarity and facilitating enhanced dipole–dipole interactions with LiOTf. Furthermore, TEGDME exhibits a higher electronegativity (χ = 0.097695 eV) compared to DME, implying superior electron-attracting capabilities and potentially stronger polarization effects on LiOTf. Despite having a slightly higher global hardness (η = 0.151365 eV), TEGDME’s overall electronic properties suggest a greater propensity for forming robust interactions with LiOTf compared to DME. Therefore, based on these considerations, it can be inferred that TEGDME likely forms stronger interactions with LiOTf in the ternary system.

**Figure 4:**
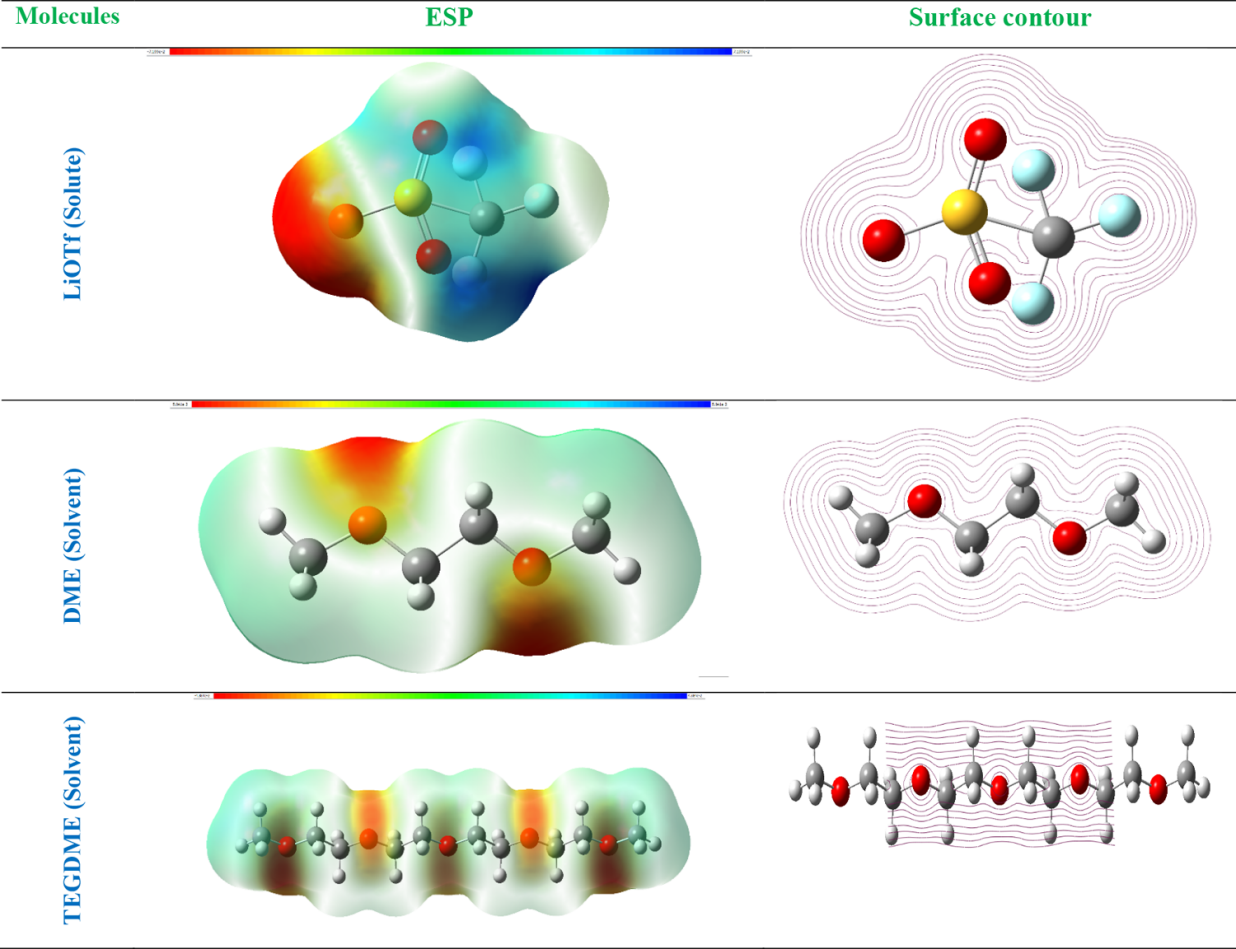
ESP and surface contour structures of the molecules present in the ternary system.

Figure 4 showcases the electrostatic potential (ESP) and surface contour structures of the molecules within the ternary system, namely, LiOTf as the solute, and TEGDME and DME as the solvents. This visualization provides valuable insights into the distribution of charge and the electrostatic environment surrounding each molecule, shedding light on their solvation behavior and potential interactions within the system. For LiOTf, the ESP map delineates regions of positive and negative ESP, indicating areas of electron deficiency and excess. This information is crucial for understanding how LiOTf interacts with the solvent molecules, as well as potential counterions or other species present in the system. The surface contour structure further illustrates the spatial distribution of charge around LiOTf, offering a visual representation of its solvation shell and potential binding sites. Similarly, the ESP and surface contour structures of DME and TEGDME provide insights into their electrostatic environments and solvation behavior within the ternary system. By examining these maps, researchers can identify regions of high and low electron density, facilitating an understanding of how the solvent molecules interact with each other and with LiOTf. Additionally, the surface contour structures offer a visual representation of the solvent molecules’ solvation shells, highlighting potential binding sites and interaction hotspots. By visualizing the ESP and surface contour structures of the molecules, researchers can gain crucial insights into their solvation behavior, intermolecular interactions, and overall performance in various applications, such as electrolytes in batteries or solvents in chemical processes. This information is essential for optimizing the design and functionality of the ternary system for specific purposes, ultimately advancing fields such as energy storage and materials science.

### 3.4 CV Studies

**Figure 5:**
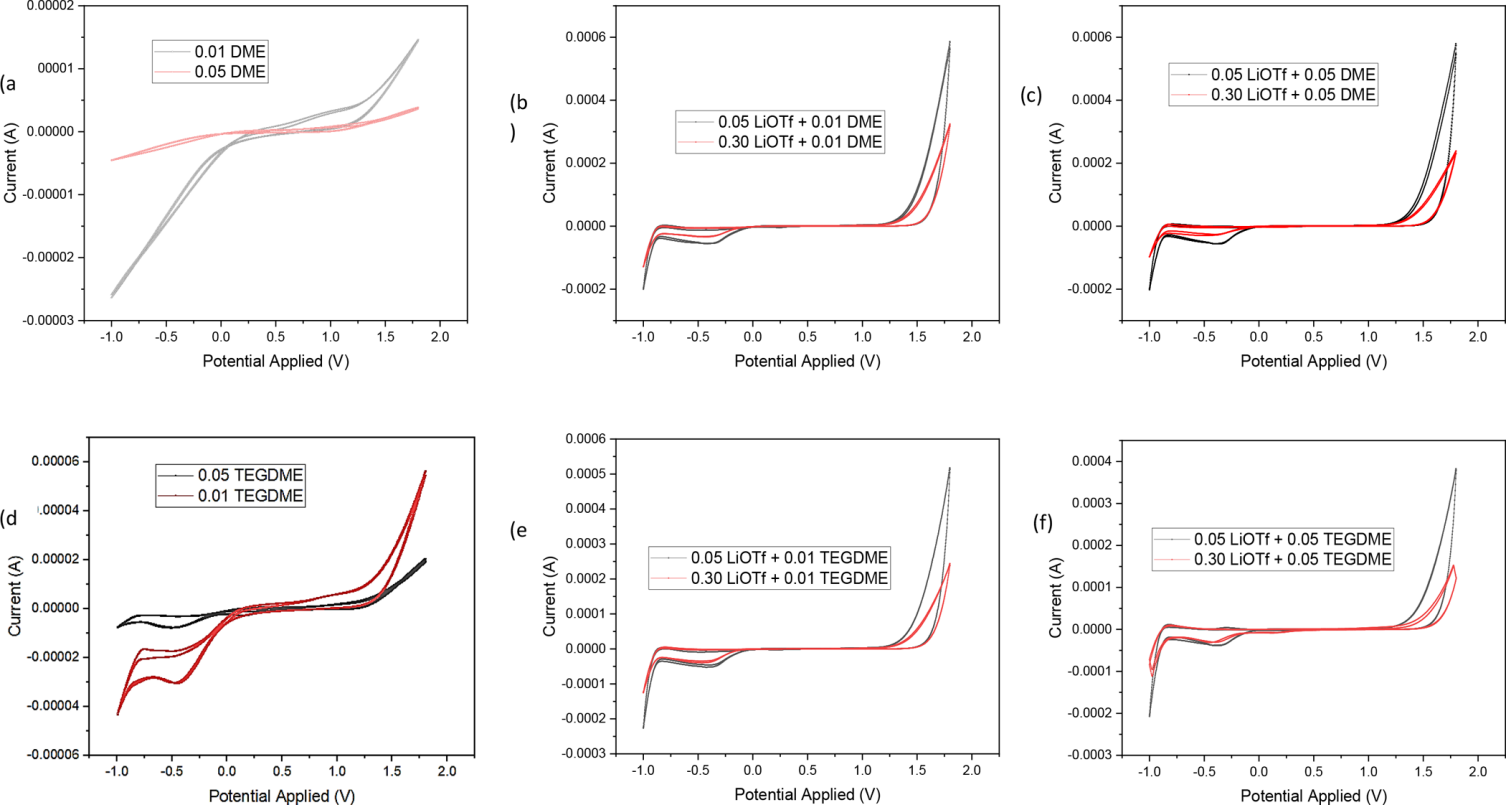
Depiction of the CV plots (V vs A) for (a) 0.01 DME and 0.05 DME, (b) 0.05 LiOTf + 0.01 DME and 0.30 LiOTf + 0.01 DME, (c) 0.05 LiOTf + 0.05 DME and 0.30 LiOTf + 0.05 DME, (d) 0.05 TEGDME and 0.01 TEGDME, (e) 0.05 LiOTf + 0.01 TEGDME and 0.30 LiOTf + 0.01 TEGDME, and (f) 0.05 LiOTf + 0.05 TEGDME and 0.30 LiOTf + 0.05 TEGDME at ambient temperature.

In this analysis, CV studies have been performed in order to determine the EW of the pure systems and systems with different concentrations of LiOTf.^56^ CV analysis was carried out at room temperature in a glovebox in an inert environment of argon flow. CV was performed on salt solutions at various concentrations and scan rates. Optimal EW values were observed at a scan rate of 100 mV/s for the solvent system containing pure DME, TEGDME, and these systems with different concentrations of LiOTf. CV graphs (Figure 5) were employed to determine the EW. These graphs plot the working electrode potential against the corresponding current.^57,58^ From the two studied systems, TEGDME was found to have a higher EW of 1.36 V in 0.01 TEGDME and 1.40 V in 0.05 TEGDME compared to that of 1.25 V in 0.01 DME and 1.38 V in 0.05 DME. Similar trends were noticed in the remaining samples, and clearly, EW also increases with an increase in the concentration of TEGDME and DME. The EW was also found to be increasing with the increasing concentration of LiOTf, as the EW of a 0.01 TEGDME system with 0.05 LiOTf is 2.23 V and with 0.30 LiOTf is 2.30 V. Similar trends were observed across the remaining samples (as shown in Table 11) with the approximate EW ranges from 2.2 to 2.32 V with different LiOTf concentrations. These data suggest promising outcomes of these studied systems, yielding favorable and comparable working EWs.^59^

**Table 11:** Several EW Values Were Attained from the CV Analysis of LiOTf in an Aqueous Solution of DME and Aqueous TEGDME at Ambient Temperature

EW (V)		
	0.01 TEGDME	0.01 DME
	1.36	1.25
0.05 LiOTf	2.23	2.20
0.30 LiOTf	2.30	2.28

	0.05 TEGDME	0.05 DME
	1.40	1.38
0.05 LiOTf	2.22	2.21
0.30 LiOTf	2.32	2.27

## 4 Conclusions

In the present work, the density and acoustic parameters for LiOTf have been evaluated in aqueous solutions of DME and TEGDME to have a better understanding of the interactions in ternary mixtures. When the results for *V*_ϕ_ and *V*_ϕ_^0^ are compared, it can be inferred that intermolecular interactions are significant in the ternary mixtures studied. The positive values of *V*_ϕ_^0^ indicated the presence of solute–solvent interactions in the ternary systems studied (LiOTf + H_2_O + DME/TEGDME). Furthermore, the values of *V*_ϕ_^0^ suggest that the (solute–solvent) interactions are more in LiOTf in DME and decrease with the concentration of DME, whereas these interactions increase as the concentration of TEGDME rises. Moreover, the positive and small negative values of Hepler’s constant confirm the structure-making tendency of the studied Li salt in DME/TEGDME. The negative values of *K*_ϕ,s_ and *K*_ϕ,s_^0^ confirm the existence of the solute–solvent interactions in the systems under investigation. Additionally, the utilization of DFT calculations has provided profound insights into the solute–solvent interactions within the ternary system composed of LiOTf as the solute and TEGDME and DME as the solvents. Quantum chemical descriptors, including *E*_HOMO_, *E*_LUMO_, IP, EA, dipole moments, electronegativity, global hardness, and chemical softness, have elucidated the electron-donating and -accepting capabilities, reactivity, and stability of the molecules, thereby highlighting their potential for solute–solvent interactions. Visualization of ESP and surface contour structures has further enhanced the comprehension of charge distribution and solvation behavior within the system. Furthermore, from the result attained from the CV analysis, TEGDME was found to have a higher EW of 1.36 V in 0.01 TEGDME and 1.40 V in 0.05 TEGDME compared to that of 1.25 V in 0.01 DME and 1.38 V in 0.05 DME. The EW was also found to be increasing with the increasing concentration of LiOTf, as the EW of a 0.01 TEGDME system with 0.05 LiOTf is 2.23 V and with 0.30 LiOTf is 2.30 V. These data suggest promising outcomes of these studied systems, yielding favorable and comparable working EWs.
